# Adding attenuation corrected images in myocardial perfusion imaging reduces the need for a rest study

**DOI:** 10.1186/1471-2342-13-14

**Published:** 2013-04-01

**Authors:** Elin Trägårdh, Sven Valind, Lars Edenbrandt

**Affiliations:** 1 Clinical Physiology and Nuclear Medicine Unit, Skåne University Hospital, Lund University, Entrance 44, Malmö, 205 05, Sweden

**Keywords:** Tc99m MPS, Ischemic cardiac disease, Attenuation correction, Stress-only studies

## Abstract

**Background:**

The American Society of Nuclear Cardiology and the Society of Nuclear Medicine conclude that incorporation of attenuation corrected (AC) images in myocardial perfusion scintigraphy (MPS) will improve diagnostic accuracy. The aim was to investigate the value of adding AC stress-only images for the decision whether a rest study is necessary or not.

**Methods:**

1,261 patients admitted to ^99m^Tc MPS were studied. The stress studies were interpreted by two physicians who judged each study as “no rest study necessary” or “rest study necessary”, by evaluating NC stress-only and NC + AC stress-only images. When there was disagreement between the two physicians, a third physician evaluated the studies. Thus, agreement between 2 out of 3 physicians was evaluated.

**Results:**

The physicians assessed 214 more NC + AC images than NC images as “no rest study necessary” (17% of the study population). The number of no-rest-study-required was significantly higher for NC + AC studies compared to NC studies (859 vs 645 cases (p < 0.0001). In the final report according to clinical routine, ischemia or infarction was reported in 23 patients, assessed as “no rest study necessary” (22 NC + AC cases; 8 NC cases), (no statistically significant difference). In 11 of these, the final report stated “suspected/possible ischemia or infarction in a small area”.

**Conclusions:**

Adding AC stress-only images to NC stress-only images reduce the number of unnecessary rest studies substantially.

## Background

Stress myocardial perfusion scintigraphy (MPS) is widely regarded as a clinically useful non-invasive imaging modality for diagnosing patients with suspected coronary artery disease. The diagnostic accuracy is high, and risk stratification has been well validated [1-3]. However, Compton scatter and depth-dependent reduction of spatial resolution degrade MPS image quality and decrease test accuracy. In addition, localized soft-tissue attenuation by the breasts, lateral chest wall, and abdomen may create artefacts that mimic true perfusion abnormalities and decrease test specificity [4,5].

Several studies have reported an increase in the diagnostic accuracy (through higher specificity) for the detection of coronary artery disease when MPS is attenuation corrected (AC) [6-12]. The American Society of Nuclear Cardiology and the Society of Nuclear Medicine conclude in their joint position statement from 2004 [6] that incorporation of AC in addition to ECG gating with MPS images will improve image quality, interpretive certainty, and diagnostic accuracy. These combined results are anticipated to have a substantial impact on improving the effectiveness of care and lowering health care costs.

One of the problems with attenuation is that it reduces laboratory efficiency by requiring comparison of stress and rest image sets to distinguish perfusion abnormalities from attenuation artefacts. If a stress study is considered completely normal, there is no need for a rest study, thus increasing laboratory efficiency and reducing the radiation dose to the patient. Heller et al. [13] investigated the clinical value of attenuation correction in stress-only MPS and found that attenuation corrected images significantly increased the ability to interpret studies as definitely normal or abnormal and reduced the need for rest imaging.

The aim of the present study was to investigate the value of adding AC stress-only images for the decision whether a rest study is necessary or not.

## Methods

### Study population

All patients admitted to Tc-99m MPS at the Department of Clinical Physiology and Nuclear Medicine, Skåne University Hospital, Malmö, Sweden in 2007 were included. In total, 1283 patients were considered. Of these, 22 patients were excluded due to missing MPS data files. The study complies with the Declaration of Helsinki and was approved by the local research ethics committee at Lund University.

### Stress-only assessment (Evaluations between NC only and NC + AC)

All stress studies were interpreted by two experienced physicians who judged each study as “no rest study necessary” or “rest study necessary”. When there was disagreement between the physicians, a third physician evaluated the studies. Thus, agreement between 2 out of 3 physicians was evaluated. The physicians evaluated two sets of images for each patient: NC images only, and both NC and AC images. The studies were blinded so that the evaluation for NC images only was not known when evaluating both NC and AC images. Stress and gated images were available for this interpretation, but not clinical information or electrocardiogram findings. The EXINI heart™ software package (EXINI Diagnostics AB, Lund, Sweden) was used to aid the interpretation.

### Clinical final report

The final reports according to clinical routine were compared to the results from the stress-only assessment described above. All cases with infarction or ischemia present or probably present were categorized as requiring a rest study, whereas all other cases were categorized not requiring a rest study. Rest (NC and AC), stress (NC and AC), gated images, clinical information, and electrocardiogram findings were available for this interpretation, which was performed according to clinical routine by the physician in charge of the study, not knowing that the patient was included in a study. In clinical routine in our department, the physician in charge evaluates the stress images, and decides whether a rest study is necessary or not. In the study population used in this study, 44% of the studies were stress-only studies. The EXINI heart™ software package was used for the visual interpretation.

### Radionuclide imaging

The MPS studies were performed using a 2-day gated stress/non-gated rest Tc-99m-tetrofosmin protocol, starting with injection of 600 MBq Tc-99m-tetrofosmin at stress. Patients were stressed using either maximal exercise on an ergometer or pharmacological test with adenosine. The exercise was continued for at least 1 min after the injection of the tracer and the adenosine infusion at least 2 min after the injection of the tracer. Normal findings at stress were not followed by a rest study. Not definitely normal stress studies were followed by a rest study with injection of 600 MBq Tc-99m-tetrofosmin. The need for a rest study was determined by the physician in charge of the study according to clinical routine.

Stress and rest acquisition began about 60 min after the end of the injection of Tc-99m-tetrofosmin. Images were obtained according to established clinical protocols, using SPECT over 180° elliptical, autocontour rotations from the 45° right anterior oblique position, with a dual-head gamma camera, e.cam (Siemens AG Medical Solutions, Erlangen, Germany). Patients were imaged in the supine position. Low energy high-resolution collimator and a zoom factor of 1.0 were used. We obtained 64 (32 views per camera) projections in a 128 × 128 matrix, with an acquisition time of 25 s per projection. Stress images were gated to the electrocardiogram using 8 frames per cardiac cycle. No automatic motion-correction program was applied; instead the acquisition was repeated if motion was detected. If motion was apparent on the repeated examination as well, a motion-correction program was used. Tomographic reconstruction and calculation of short and long axis slice images were performed using e.soft (Siemens AG Medical Solutions, Erlangen, Germany). NC images were reconstructed with filtered back-projection. A 2D Butterworth pre-reconstruction filter was used with critical frequency of 0.45, order 5. AC images were reconstructed with an iterative algorithm, 6 iterations [14] where a ramp filter was applied on the error projection prior to backprojection. A Butterworth filter with a cut-off frequency of 0.40, order 5, was applied for regularization. Attenuation maps were generated from simultaneous transmission measurement using a Gd-153 multiple-line source (Siemens AG Medical Solutions, Erlangen, Germany) [15].

### Statistical analysis

The McNemar test was used to analyze the differences in classification of patients between the NC only group and the NC + AC images group. The level of statistical significance was set at 0.05. Analyses were carried out using Analyse-it® for Microsoft Excel (Analyse-it Software Ltd, Leeds, UK).

## Results

The number of no-rest-study-required cases was significantly higher using the NC + AC studies compared to the NC studies (859 vs 645 cases (p < 0.0001). In 634 cases (50%) the evaluations for both NC and NC + AC stress images were no-rest-study-required and in 391 cases (31%) that a rest study was required. In 11 cases (1%), the physicians did not want a rest study when evaluating NC images, but wanted a rest study when evaluating NC + AC images. In 225 cases (18%), the physicians thought it was necessary to perform a rest study when evaluating NC images, but not when evaluating NC + AC images.

961 studies (76%) were considered normal (i.e. no ischemia or infarction present) based on the final report according to clinical routine. In 8 of the 645 (1.2%) NC stress images classified as no-rest-study-required, ischemia/infarction was present on the final report. The corresponding results for the NC + AC images were 22 of 859 (2.6%) and this difference was not statistically significant. Cases classified as rest-study-required were more often interpreted as ischemia/infarction in the final report for the NC + AC images (69%) than the NC images (47%) (p < 0.0001). The numbers are shown in Table 1A and B.

**Table 1:** The distribution of the evaluations from NC images (A) and NC + AC images (B)

			**Final report**	
		**Normal**	**Abnormal**	
A				
NC only	No rest	637	8	**645**
	Rest	324	292	**616**
		**961**	**300**	**1261**
B				
AC + NC	No rest	837	22	**859**
	Rest	124	278	**402**
		**961**	**300**	**1261**

### Patients with abnormal MPS (final report) that were missed on stress-only images

In total, 23 patients had ischemia and/or infarction on the final reports, but were regarded as “no-rest-study-required” on either NC or NC + AC stress image evaluation; thus would falsely have been sent home without a rest study. Of these, 1 patient was missed when evaluating NC images, 15 when evaluating NC + AC images and 7 patients were missed for both evaluations. In 11 of the 23 cases, the final report stated “suspected/possible ischemia or infarction in a small area of the left ventricle”; in 9 cases “ischemia or infarction in a small area of the left ventricle”; and in 3 cases “ischemia in a moderately large area of the left ventricle”. Two of the patients with moderately large ischemia underwent coronary angiography within 6 months after the MPS, and both received percutaneous coronary intervention (PCI). The third patient underwent coronary angiography with PCI of the right coronary artery 4 years after the MPS. Three of the 9 patients with “ischemia/infarction in a small area” underwent coronary angiography; two without any need of PCI and one with PCI of the left ascending coronary artery and the left circumflex artery. Only 1 patient with “suspected ischemia” underwent coronary angiography, with subsequent PCI of a diagonal branch. This patient got a dissection in the left main coronary artery during the angiography.

## Discussion

In the present study we investigated the accuracy for determining the need for a rest study when using NC images or NC + AC images. We found that adding AC images were superior to using only NC images. When using NC + AC compared to NC images, 214 more patients (17% of the study population) could avoid a rest study. The advantages of being able to reduce the number of rest studies are substantially reduced radiation exposure, lower costs by eliminating unnecessary imaging time and radiopharmaceutical doses, and improved laboratory efficiency by freeing up camera time to study additional patients.

In this material, when using NC images, 1.2% of the patients would have been sent home without a rest study, when a rest study was needed according to the final report. The number for AC images was 2.6% (no statistically significant difference). The reasons for this could be either that the final report was not always correct or that the physicians who evaluated the stress studies did not have access to the clinical information (as stated in the study limitations section) or that can be difficult to see small perfusion abnormalities on stress-only images in some cases. The number of missed certain ischemia/infarction due to a normal interpretation on stress-only images was very low in this study (less than 1%), but the goal is not to miss any patients with ischemia or infarction. In a study by Johansson et al. [16], trying to decide whether nuclear medicine technologists are able to determine the necessity for a rest study, 2.6% of the patients would have been sent home without a rest study, when a rest study was needed according to gold standard (ischemia or infarction on combined stress-rest interpretation). Thus, their results were similar to the ones found in the present study.

The stress-only approach has been investigated in several studies. Worsley et al. [17] demonstrated that rest images were not required if normal imaging findings had been obtained after exercise or pharmacologic stress. This was later confirmed by Schroeder-Tanka et al. [18] in a larger study. Heller et al. [13] found that AC applied to studies with stress-only ^99m^Tc MPS significantly increased the ability to interpret studies as definitely normal or abnormal and reduced the need for rest imaging. In their study, ten experienced nuclear cardiologists independently interpreted 90 stress-only MPS in a sequential fashion: MPS alone, MPS plus ECG-gated data, and AC MPS with ECG-gated data. Images were interpreted for diagnostic certainty (normal, probably normal, equivocal, probably abnormal, abnormal, and perceived need for rest imaging). Adding AC data increased the number of studies characterized as definitely normal or abnormal to 84% (37% for MPS data alone) and reduced the perceived need for rest imaging from 77% for MPS data only to 43% for the addition of AC data. Heller et al., however, included far fewer patients than the present study, and only included patients with either known coronary artery disease or patients with a 5% or lower likelihood of coronary artery disease. The ten independent readers also interpreted the studies in a sequential manner. The fact that only patients with known coronary artery disease or patients with a very low likelihood of coronary artery disease in combination with the sequential interpretation could be the reason for the larger difference in the number of rest studies needed when adding AC images compared to NC images only found in their study compared to the present study.

Current guidelines also recommend the stress study to be performed first, since the rest study can be omitted if the stress study is interpreted as normal [19]. Thus a rest study should only be performed in patients with equivocal or clearly abnormal studies. Chang et al. [20] investigated whether a normal stress-only MPS confers the same prognosis as a normal MPS on the bases of evaluation of stress and rest images. They found that patients who had a normal MPS on the basis of stress imaging alone have a similar mortality rate as those who have a normal MPS on the basis of evaluation of both stress and rest images.

### Study limitations

In the present study the final reports according to clinical routine was used to compare the results from the stress-only assessment by the three study physicians. This might not be the optimal since physicians with different levels of experience interpreted the studies, and because there is always inter-observer variability when interpreting studies.

The physicians who evaluated the NC and AC stress studies did not have any clinical information about the patients. It is possible that fewer patients would have been falsely regarded as “no-rest-study-required” if the clinical information was available, as it is in the true clinical setting.

A third limitation is that in clinical routine at our department, physicians already evaluate the need for a rest study after the stress study (based on NC and AC stress images, gated images and clinical information). In 44% of the cases, no rest study is performed, and “no ischemia or infarction” is stated on the final report. If any mistakes were done in the clinical assessment, the final report used in the present study could be wrong.

## Conclusion

Adding AC stress-only images to NC stress-only images reduce the number of unnecessary rest studies substantially. The risk that a patient sent home without a rest study would have been diagnosed with definite infarction or ischemia using the combined stress-rest interpretation was low. 214 more patients (17% of the study population) did not require a rest study when adding AC images. We conclude that adding AC images will improve laboratory efficiency.

## Competing interests

LE is employed by and stockholder of EXINI Diagnostics.

## Authors’ contributions

ET participated in the design of the study, acquired and evaluated the data, performed the statistical analysis and drafted the manuscript. SV evaluated the data and helped to draft the manuscript. LE participated in the design of the study, evaluated the data and helped to draft the manuscript. All authors read and approved the final manuscript.

## Pre-publication history

The pre-publication history for this paper can be accessed here:

http://www.biomedcentral.com/1471-2342/13/14/prepub
